# The Mechanistic Differences in HLA-Associated Carbamazepine Hypersensitivity

**DOI:** 10.3390/pharmaceutics11100536

**Published:** 2019-10-15

**Authors:** Gwendolin S. Simper, Lareen S. Gräser, Alexander A. Celik, Joachim Kuhn, Heike Kunze-Schumacher, Gia-Gia T. Hò, Rainer Blasczyk, Andreas Pich, Christina Bade-Doeding

**Affiliations:** 1 Institute for Transfusion Medicine, Hannover Medical School, Carl-Neuberg-Str. 1, 30625 Hannover, Germany; Simper.Gwendolin@mh-hannover.de (G.S.S.); lareen.graeser@mpibpc.mpg.de (L.S.G.); alexander.celik@outlook.com (A.A.C.); Heike.Kunze-Schumacher@kgu.de (H.K.-S.); Ho.Gia-Gia@mh-hannover.de (G.-G.T.H.); Blasczyk.Rainer@mh-hannover.de (R.B.); 2 Institute for Laboratory and Transfusion Medicine, Heart and Diabetes Center North Rhine-Westphalia, Ruhr University Bochum, Georgstraße 11, 32545 Bad Oeynhausen, Germany; JKuhn@hdz-nrw.de; 3 Institute of Toxicology, Hannover Medical School, Carl-Neuberg-Str. 1, 30625 Hannover, Germany; Pich.Andreas@mh-hannover.de

**Keywords:** adverse drug reaction, HLA-A*31:01, HLA-B*15:02, carbamazepine, carbamazepine-10,11-epoxide, proteome

## Abstract

Drug hypersensitivity reactions that resemble acute immune reactions are linked to certain human leucocyte antigen (HLA) alleles. Severe and life-threatening Stevens Johnson Syndrome and Toxic Epidermal Necrolysis following treatment with the antiepileptic and psychotropic drug Carbamazepine are associated with HLA-B*15:02; whereas carriers of HLA-A*31:01 develop milder symptoms. It is not understood how these immunogenic differences emerge genotype-specific. For HLA-B*15:02 an altered peptide presentation has been described following exposure to the main metabolite of carbamazepine that is binding to certain amino acids in the F pocket of the HLA molecule. The difference in the molecular mechanism of these diseases has not been comprehensively analyzed, yet; and is addressed in this study. Soluble HLA-technology was utilized to examine peptide presentation of HLA-A*31:01 in presence and absence of carbamazepine and its main metabolite and to examine the mode of peptide loading. Proteome analysis of drug-treated and untreated cells was performed. Alterations in sA*31:01-presented peptides after treatment with carbamazepine revealed different half-life times of peptide-HLA- or peptide-drug-HLA complexes. Together with observed changes in the proteome elicited through carbamazepine or its metabolite these results illustrate the mechanistic differences in carbamazepine hypersensitivity for HLA-A*31:01 or B*15:02 patients and constitute the bridge between pharmacology and pharmacogenetics for personalized therapeutics.

## 1. Introduction

Adverse drug reactions (ADRs) are harmful reactions to appropriately dosed drugs despite proper application (WHO 1972). Although they cause morbidity and mortality those complications are underdiagnosed, underreported and raise high costs [1,2,3,4,5]. ADRs are subdivided into dose-dependent predictable type A ADRs elicited by the pharmacological activity of the drug and idiosyncratic type B ADRs [6,7]. While the majority of all ADRs is classified as rarely fatal type A reaction with clinical symptoms depending on the mode of action of the drug, it has been found that severe type B reactions are often immune-mediated and sometimes associated with certain alleles of the human leucocyte antigen (HLA) system [2,8,9].

In their function of presenting peptides to immune effector cells HLA molecules are characterized by the amino acids (AAs) forming the peptide binding region (PBR). The immune recognition of self/nonself is facilitated by presenting peptides of intracellular origin to CD8^+^ T cells; each HLA class I variant exhibits a PBR composed of pockets interacting with the side chains of bound peptides [10,11]. The AA composition of the PBR is allele specific and therefore determines peptide-binding motifs [12,13,14]. The peptide loading complex, composed of the HLA heavy and light chain, the chaperones calreticulin and tapasin (TPN) and the thiol oxidoreductase ERp57, enables loading of high affinity peptides, even though TPN-independent loading of low affinity peptides is possible [15,16]. The T cell receptor (TCR) of CD8^+^ cells recognizes subtle AA alterations of the bound peptides and appreciates simultaneously host HLA class I bound to peptides of self or foreign origin. In that way, the TCR scans the intracellular proteome continuously to verify the health status of a cell.

Since most synthetical drugs are of small size (<1 kDa) they are invisible to the immune system themselves. By binding to a carrier protein, a reactive drug (hapten) or a drug (prohapten) that acts as a hapten post-metabolization can become immunogenic and trigger an immune reaction through several mechanisms. According to Gell and Coombs, the hapten-protein complex might be recognized by IgE antibodies leading to the activation of mastcells and basophils in type I reactions, IgG antibody-dependent cell-mediated cytotoxicity in the case of type II reactions or the formation of immune-complexes in type III reactions [17,18]. Moreover, drugs are able to provoke a T-cell mediated type IV reaction. For those type IV reactions, an association with HLA class I molecules can be explained by their etiology: After processing of the hapten-protein complex a modified peptide is presented by a certain HLA molecule leading to the recognition of nonself (hapten model) as occurring in hypersensitivity reactions to beta-lactam antibiotics [19,20,21,22]. Alternatively, the drug may non-covalently bind to the HLA molecule and/or the TCR at their interface interfering with the highly accurate interaction between the cell surface molecules (pharmacological interaction (p-i) model) [20,23]. As described for the anti-retroviral drug abacavir in HLA-B*57:01-positive patients, binding of the drug to the PBR alters its biophysical and biochemical properties and consequently leads to the selection and presentation of an altered peptide repertoire that is recognized as foreign by the host immune system, resulting in the induction of an (auto) immune response (altered repertoire model) [24,25]. Presentation of an altered peptide repertoire that is not significantly distinguished on the basis of their peptide binding motifs can certainly trigger a strong CD8^+^ T cell reaction. This could e.g., be described for the allelic variants B*44:02 and B*44:03 that are discriminated by a single amino acid (AA) exchange in the peptide binding region (PBR) [26].

Primarily used to treat partial onset seizures, but also applied in case of trigeminal neuralgia and bipolar disorders, the tricyclic anticonvulsant and psychotropic drug carbamazepine (CBZ) can induce cutaneous ADRs in patients as reported soon after the first prescriptions [27,28,29,30,31,32].

The clinical symptoms of patients suffering from CBZ-mediated ADRs range from milder skin exanthemas to severe and life-threatening syndromes [33]. They comprise maculopapular exanthema (MPE) where patients exhibit a rash with macules or erythematous and maculopapular lesions as well as cutaneous ADRs involving multiple organs. As such, in drug reaction with eosinophilia and systemic symptoms (DRESS) in addition to cutaneous exanthema variable features are observed including changes in blood cell count, hepatitis, arthralgia and lymphadenopathy [34]. Stevens-Johnson syndrome (SJS) and toxic epidermal necrolysis (TEN) with a lethality of 48% are life-threatening complications of CBZ-medication [35]. They are characterized by fever, hypovolemia, detachment of the skin, formation of blisters and bullae, as well as erosions of mucous membranes. In SJS the skin detachment comprises no more than 10% of the body surface area, whereas in TEN the skin of at least 30% of the body surface area has to be detached [35,36].

A strong association of CBZ-mediated SJS/TEN with the allele HLA-B*15:02 was found in the Han Chinese population [37]. This allele is mainly prevalent in South Asia, whereas it is extremely rare in Europe with an allele frequency of less than 0.01% [38]. Therefore, the association of CBZ-mediated SJS/TEN with HLA-B*15:02 could not be confirmed for Caucasians [39], but for other South East Asian ethnicities [40,41,42,43,44,45]. These findings have led to the recommendation of the US food and drug administration to screen patients with an Asian background for the presence of HLA-B*15:02 before administration of CBZ [46].

Interestingly, the allele HLA-A*31:01 has also been described to be associated with several cutaneous ADRs after administration of CBZ in various demographic groups, such as, for example Caucasians, Japanese and Koreans [47,48,49]. HLA-A*31:01 is distributed worldwide, with an allele frequency between 2.1% and 3.6% in Europe, up to 12% in Japan and maxima in Argentina (25–38.6%) and Brazil (2.6–18.5%) [38].

Both HLA alleles differ substantially in their AA composition and their immune function from each other. The peptide binding motif of HLA-B*15:02 has been described to be a nonpolar aromatic pΩ anchor [24,50,51], whereas peptides presented by HLA-A*31:01 are preferentially anchored by Arginine at pΩ [52]. Likewise, a distinct discrepancy between the clinical outcome of HLA-B*15:02 or A*31:01 positive patients following CBZ administration has been described. Carriers of HLA-B*15:02 can solely develop severe SJS/TEN, whereas HLA-A*31:01-carriers show a wider area of symptoms including MPE or DRESS [38,40,47]. The median onset of CBZ-mediated DRESS in the RegiSCAR study was 29 days, whereas CBZ-mediated SJS/TEN emerged after 15 days [34]. These differences in clinical symptoms imply that the molecular mechanisms of the ADRs might diverge from each other.

In HLA-B*15:02-positive patients suffering from CBZ-mediated ADRs CBZ-specific CD8^+^ T cells were found and analyzed for V_ß_ chain of the TCR leading to the identification of a restricted TCR usage [53].

CBZ degradation to its main metabolite CBZ-10, 11-epoxide (EPX) is mediated by cytochrome P450 enzymes, mainly CYP3A4 [40]. EPX is detoxified by epoxide hydroxylases to CBZ-10, 11-trans-diol which is urinarily secreted [54]. In Han Chinese population it was shown that CBZ-induced ADRs are not associated with polymorphisms in P450 enzymes [37]. For HLA-B*15:02 it has been described that treatment with CBZ does not significantly alter the peptidome [24,50,51]. Since not only CBZ but also its metabolites, including EPX, are present in the body, it is not necessarily CBZ that triggers the ADR. Wei et al. have demonstrated that derivates of CBZ are able to bind soluble immobilized HLA-B*15:02 as well [55]. In fact, EPX is not only binding to the F pocket of HLA-B*15:02 but is also altering the peptide binding motive of the presented peptides [51]. This is consistent with the finding that the risk of SJS/TEN depends on polymorphisms of the epoxide hydroxylases 1 (EPHX1) in Han Chinese population: The variant c.337T > C in EPHX1 was associated with an increased risk of SJS/TEN after administration of CBZ [56].

Less is known regarding HLA-A*31:01-associated CBZ hypersensitivity. CBZ-specific HLA-A*31:01-restricted CD8^+^ T cells as well as HLA-DRB1*04:04-restricted CD4^+^ T cells were identified among CBZ-reactive T cell clones derived from an HLA-A*31:01-positive patient with CBZ hypersensitivity. The data led to the assumption of an influence of the HLA haplotype on a multiclonal T cell response [57]. Until now, the effect of CBZ and EPX treatment on HLA-A*31:01 has not been analyzed.

**Figure 1:**
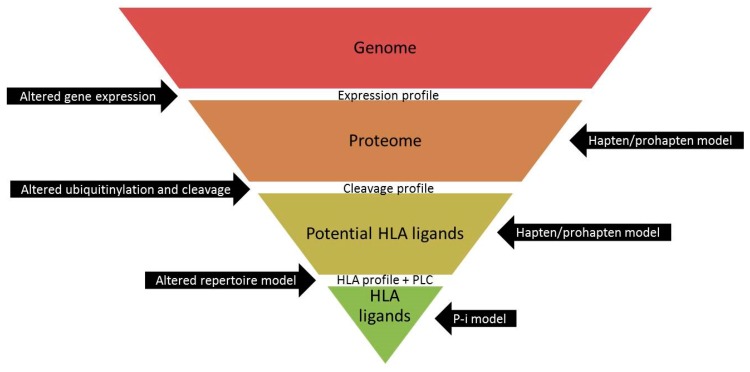
Filters to be passed before a human leucocyte antigen (HLA) ligand is presented by a HLA molecule. Opportunities to influence peptide presentation are indicated in the black arrows.

The aim of this work was to comprehend the differences in HLA-B*15:02 and HLA-A*31:01-associated CBZ-mediated ADRs. Therefore, we mass spectrometrically examined the ability of CBZ and EPX to alter peptide presentation of HLA-A*31:01. Additionally, we compared peptide loading mode of HLA-B*15:02 and HLA-A*31:01 and analyzed the effect of drug treatment on the proteome, since there might be more reasons for the diverging clinical pictures in HLA-B*15:02 and HLA-A*31:01-positive patients suffering from CBZ-mediated ADRs (Figure 1). Drug treatment might influence the expression of proteins, leading to an altered pool of potentially presented peptides. Alternatively, the process of peptide loading might be varied through changes in expression levels of an involved protein.

Clarifying the differences in HLA-B*15:02 and HLA-A*31:01-associated CBZ-mediated ADRs on a molecular level will be a step forward to facilitate personalized and safe treatment of patients to prevent life-threatening diseases.

## 2. Materials and Methods

### 2.1. Maintenance of Cell Lines

All cell lines were cultured at 37 °C and 5% CO_2_. The human B-lymphoblastoid cell lines LCL721.220 [58] (LGC Promochem, Wesel, Germany; HLA class I^−^/TPN^−^) and *LCL721.221* [59] (LGC Promochem, Wesel, Germany; HLA class I^−^/TPN^+^) were grown in RPMI 1640 (Lonza, Basel, Switzerland) supplemented with 10% heat inactivated fetal calf serum (FCS, Lonza, Basel, Switzerland), 2 mM l-glutamine (c. c. pro, Oberdorla, Germany), 100 U/mL penicillin and 100 µg/mL streptomycin (c. c. pro).

For the human embryonal kidney cell line HEK293T [60] (Thermo Fisher Scientific, Rockford, IL, USA), DMEM (Lonza) supplemented with 10% heat inactivated FCS, 2 mM l-glutamine, 100 U/mL penicillin, 100 µg/mL streptomycin and 1 mg/mL Geneticin^®^ (Life Technologies, Carlsbad, CA, USA) was utilized as medium.

### 2.2. Production of Soluble HLA Molecules

Soluble HLA (sHLA) molecules were expressed in human B-lymphoblastoid cell lines *LCL721.220* (HLA class I^−^/TPN^−^) and *LCL721.221* (HLA class I^−^/TPN^+^). Cloning of the lentiviral vector encoding for sHLA-B*15:02 (exon 1–4) [51] and sHLA-A*31:01 (exon 1–4), respectively, has been previously described [61].

For generation of lentiviral particles, HEK293T cells were transfected with the target plasmid pRRL.PPT.SFFV.mcs.pre/sA*31:01 or pRRL.PPT.SFFV.mcs.pre/sB*15:02 (10 µg/5 × 10^6^ cells) together with the packaging and envelope vectors *psPAX2* and *pmD2.G* (each 5 µg/5 × 10^6^ cells) using Lipofectamine^®^ 2000 (Life Technologies, Carlsbad, CA, USA) as described by Bade-Doeding et al. [62]. Following 8 h incubation the medium was exchanged. 36 h posttransfection, virus-containing supernatant was passed through a 0.45-µm filter (Millipore GmbH, Schwalbach, Germany) and concentrated overnight by centrifugation at 16 °C at 10.000 rpm. The lentiviral pellet was dissolved in RPMI 1640. Transduction of B-lymphoblastoid cell lines was performed by adding the virus concentrate in the presence of 8 µg/mL protamine sulfate (Sigma-Aldrich, St. Louis, MO, USA) to 5 × 10^5^ cells. Following 8 h incubation, cells were cultured in complete RPMI 1640 medium. Successful transduction of *LCL721.220* cells and *LCL721.221* cells was verified by detection of trimeric sHLA molecules in the cell culture supernatant via an HLA class I-specific ELISA [62,63].

The antibody w6/32 was used as capture antibody; an anti-β2m (Dako, Santa Clara, CA, USA) and an anti-rabbit HRP-conjugated (Dako, Santa Clara, CA, USA) antibody served as detection antibodies. TMB One^TM^ substrate (KEM-EN-Tec Diagnostics, Taastruo, Denmark) was employed for the substrate reaction according to Celik et al. [63].

The resulting cell lines have been cultured at a cell density of 1 × 10^6^ cells/mL with or without 25 µg/mL CBZ or EPX (both Toronto Research Chemicals, Toronto, ON, Canada) for production of sHLA-B*15:02 (sB*15:02) and sHLA-A*31:01 (sA*31:01) complexes, respectively, in absence or presence of the drugs according to Simper et al. [51]. The supernatant containing sHLA molecules was collected twice a week. Cells and cellular debris were discarded by centrifugation. Additionally, supernatant was filtered through a 0.45-µm membrane and adjust to pH 8.0. sHLA-A*31:01 (w/o drug, w/CBZ, and w/EPX) complexes were purified via affinity chromatography using an NHS-activated HiTrap column (Life Technologies, Carlsbad, CA, USA) coupled to an anti-HLA class I antibody (clone W6/32). Elution of molecules was performed with 100 mM glycine/HCl buffer pH 2.7.

### 2.3. Mass Spectrometric Sequencing of the Presented Peptides and Measurement of the Drugs

Mass spectrometric sequencing of peptides eluted from those functional sHLA complexes and detection of CBZ and EPX has been performed according to Simper et al. [51].

### 2.4. Mass Spectrometric Analysis of Drug-Induced Modifications of the Proteome

For proteome analysis, cells were lysed in RIPA buffer as described by Ho et al. [64]. Cell suspension was thoroughly vortex and incubated on ice for 30 min. Following centrifugation (15 min, 13,000 rpm, 4 °C), the supernatant containing the protein was harvested.

The amount of protein was ascertained by photometrical measurements according to Lowry et al. [65] using the DC™ Protein Assay kit (Bio-Rad Laboratories, Hercules, CA, USA). With a SmartSpec™ 3000 Photometer (Bio-Rad Laboratories, Hercules, CA, USA) the absorption of the samples was measured at 750 nm.

Digestion in solution was performed as modified version called filter aided sample preparation (FASP) method, adapted from Wiśniewski et al. [66]. Samples were adjusted to 25 mM DTT (Sigma Aldrich Co. LLC, St. Louis, MO, USA) and denaturized at 50 °C for 45 min. Urea buffer (pH 8.5; 8 M; Sigma Aldrich Co. LLC, St. Louis, MO, USA) was added to 300 μg of protein. Proteins were bound to a centrifugal filter by centrifugation at 14,000× *g* for 15 min. The free cysteines were carbamidomethylated by incubating in 0.05 M iodoacetamide in urea-buffer for 20 min in the dark. The bound proteins were washed first with urea-buffer and then 40 mM ammoniumbicarbonate (ABC; Sigma Aldrich Co. LLC, St. Louis, MO, USA) for 15 min at 14,000× *g*. After digestion of the proteins with trypsin solution (1 μg/µL trypsin (SERVA Electrophoresis GmbH, Heidelberg, Germany) in 40 mM ABC) at 37 °C overnight and by centrifugation at 14,000× *g* for 10 min the flow through was collected. Remaining peptides were eluted with ABC and ABC with 15% acetonitrile (ACN; Carl Roth GmbH & Co. KG, Karlsruhe, Germany). Finally, trifluoroacetic acid (TFA 10%; J.T. Baker, Phillipsburg, NJ, USA) was added and the collected run-through samples were dried in a vacuum centrifuge.

Samples were fractionated in order to reduce complexity and thereby increase the amount of identified proteins. Prior to the LC-MS analysis, peptides were separated by hydrophobicity via high pH reversed-phase chromatography applying the Pierce High pH Reversed-Phase Peptide Fractionation Kit (Thermo Fisher Scientific, Rockford, IL, USA). Elution solutions were prepared according to Table 1. Liquid contents of each fraction were evaporated to dryness.

For LC-MS analysis peptides were dissolved in 2% ACN and 0.1% TFA at 850 rpm for 30 min at RT. The solution was centrifuged at 13,000 rpm for 5 min and transferred into an LC-vial for LC-MS analysis.

For LC-MS analysis, a nanoflow ultrahigh performance liquid chromatography (Dionex UltiMate 3000 Rapid Separation LC (RSLC) System) coupled to an LTQ Orbitrap Lumos mass spectrometer was used [67]. Sample solutions were first loaded on a trap column (3 μm C18 particles, 2 cm length, 75 μm ID, Acclaim PepMap (Thermo Fisher Scientific, Rockford, IL, USA) before the peptides were separated on a reversed phase column (50 cm, 2 μm C18 particles, 75 μm ID, Acclaim PepMap (Thermo Fisher Scientific, Rockford, IL, USA). Peptide were eluted by a 70 min binary multistep gradient (solvent A: 0.1% FA solvent B: 80% ACN, 0.1% FA) with a flow rate of 250 nL/min (see Figure S1).

MS1 scans from *m*/*z* = 375 to 1500 were measured in the Orbitrap mass analyzer with automatic gain control set to 4 × 105 and maximum injection time set to 50 ms. Resolution of the Instrument was set to 120,000 at *m*/*z* = 200. Most intense precursors were selected in the MS surveillance scan (“TopX”-method) for collision induced dissociation (CID)-fragmentation and analysis in the ion trap. Normalized collision energy of 35% was used. For MS2 fragmentation an automatic gain control target of 3 × 104 was set and maximum injection time was set to 300 ms. To prevent the reoccurring fragmentation of very intense ions a dynamic exclusion list was created that prevents those ions to be repeatedly isolated and fragmented within 10 s after second occurrence.

The MS/MS-spectra were analyzed using the MaxQuant software with the implemented Andromeda database search [68]. The proteomic data were further processed with the Perseus software (version 1.6.1.3) [69]. As recommended, records containing reverse and potential contaminant were filtered. Intensities were given as log values to base 2. Protein groups detected in every replicate were classified as quantifiable. Missing values were imputed according to normal distribution. Each protein was annotated based on the GOBP-, GOMF-, GOCC- and KEGG-database. Proteins were briefly characterized based on Gene Ontology data from the UniProt human database [70]. Then, 95% confidence range regulation limits were specified for all LFQ-based proteome analyses on the basis of the variance between the biological replicates of the control proteomes.

Network analyses were conducted by the Ingenuity Pathway Analysis (release June 2018) using recommended parameters for the Core Analysis [71]. Networks were further manually edited.

## 3. Results

### 3.1. HLA-A*31:01 and B*15:02 Differ Functionally by Their Peptide Loading

To evaluate if A*31:01 and B*15:02 acquire their peptides through the chaperoning of TPN, *LCL721.221* and *LCL721.220* cells were transduced with constructs encoding for sA*31:01 and sB*15:02 molecules. An HLA class I-specific ELISA was utilized to analyze the presence of functional sHLA molecules. A*31:01 was able to be assembled and secreted in the absence of TPN, while B*15:02 revealed a stringent TPN dependency for peptide selection and presentation (see Figure S2).

### 3.2. HLA-A*31:01 Shows a Preference for Presentation of 9 and 11 Mers

The human lymphoblastoid cell lines *LCL721.220* and *LCL721.221* were successfully transduced with vectors encoding for sA*31:01. Following affinity purification of the trimeric peptide-HLA (pHLA) complexes low and high binding peptides were separated and sequenced as previously described [51].

**Figure 2:**
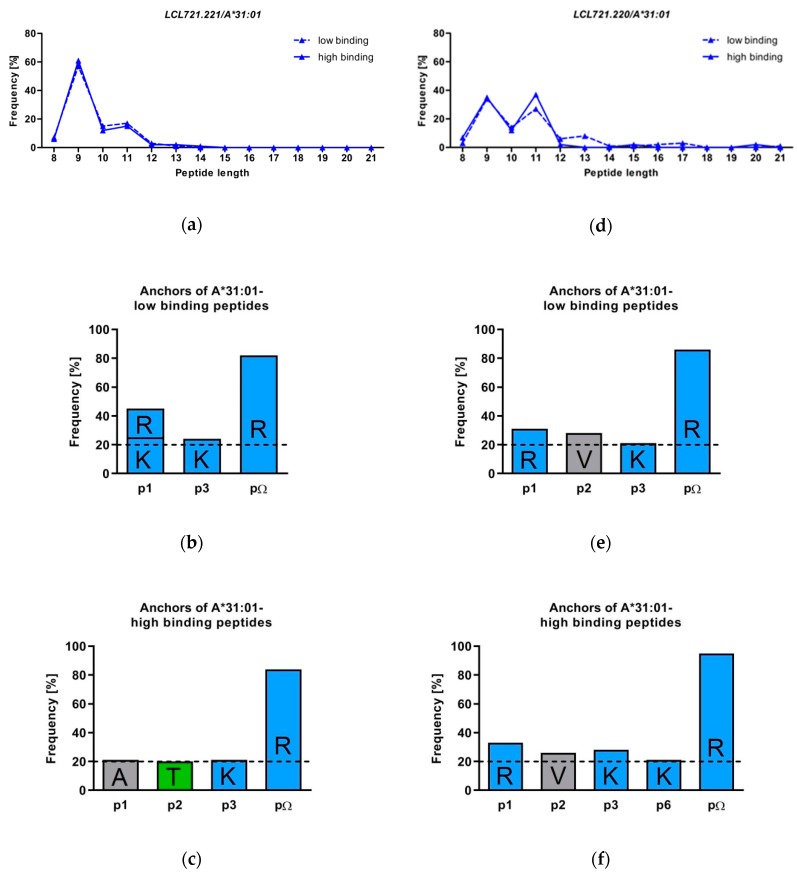
Analysis of the peptide repertoire of sA*31:01 (**a**) amino acids (AA) length of peptides presented by sA*31:01 in *LCL721.221* cells; (**d**) AA length of peptides presented by sA*31:01 in TAP-deficient *LCL721.220* cells. Peptides are distinguished into low binding and high binding peptides; (**b**,**c**) Peptide anchors of sA*31:01 low and high binding peptides produced in *LCL721.221* cells; (**e**,**f**) Peptide anchors of sA*31:01 low and high binding peptides produced in *LCL721.220* cells. The single letter code is used for AAs. Polar positive (blue), polar neutral (green), nonpolar aliphatic (gray) and nonpolar aromatic (purple) AAs with a frequency above 20% (dashed line) are colored in the same color.

The peptides presented by A*31:01 in *LCL721.221* cells were 8 to 14 AAs in length (Figure 2a). 9-meric peptides were found most frequently, although the amount of 11-mers was augmented for A*31:01 both in low and high binding peptide fractions. This length preference of A*31:01 is consistent with the findings of Abelin et al. [52].

Peptides with an AA length ranging from 8 to 21 were eluted from sA*31:01 expressed in *LCL721.220* cells (Figure 2b). The amount of 11 mers was increased in this cell line as well, low binding peptides had mostly a length of 9 (34%), 11 (27%) or 10 AAs (14%). High binding peptides were found most frequent as 11 mers (37%), followed by 9 mers (35%) and 10 mers (12%).

### 3.3. The Peptide Binding Motif of HLA-A*31:01 Exhibits a Strong Arginine Anchor at pΩ

In *LCL721.221* cells expressing sA*31:01 82% of all low binding and 84% of all high binding peptides were anchored by Arginine at pΩ (Figure 2c,d). Both low and high binding peptides shared Arginine as pΩ anchor, yet, the peptides differed at other amino acid positions within the peptide sequence. HLA-A*31:01-low binding peptides showed a high frequency of the polar and positively charged AAs Lysine (25%) and Arginine (20%) at p1, whereas in high binding peptides Alanine (21%) was most frequent. For low binding peptides, no distinct peptide anchor could be defined at p2. In contrast, A*31:01-high binding peptides preferred Threonine (20%) at this position. Of note, Lysine was most frequent at p3 for both low and high binding peptides (24% and 21%). Low and high binding sA*31:01-restricted peptides derived from *LCL721.220* cells were comparable to low binding peptides derived from *LCL721.221* cells. Those peptides exhibited the Arginine anchor at pΩ with a frequency of 95% in high binding peptides and 86% in low binding peptides (Figure 2e,f). Polar and positively charged Arginine dominated p1 with 31% low and 33% in high binding peptides. For both A*31:01-low and high binding peptides Valine was most frequent (28% and 26%) at p2. The preference for polar positive Lysine at p3 was also shared by low (21%) and high (28%) binding peptides in *LCL721.221* cells. In high binding peptides Lysine was found with a frequency of 21% at p6. The auxiliary anchor Threonine at p2 found in high binding peptides derived from *LCL721.221* cells was not observed with the same frequency in peptides derived from *LCL721.220* cells.

### 3.4. Treatment with CBZ Alters the Motif of HLA-A*31:01 High Binding Peptides

**Figure 3:**
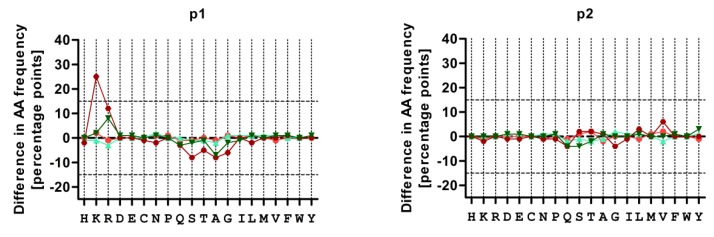

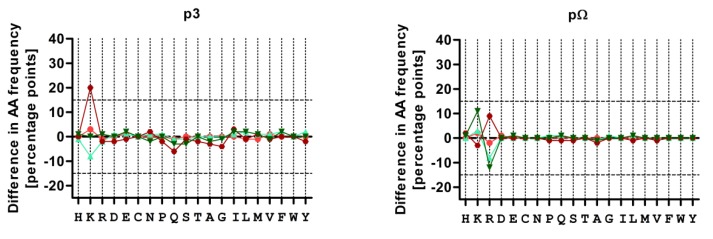
Incubation with carbamazepine (CBZ) provokes a shift in high binding peptides bound to sA*31:01. Changes in AA frequencies of sA*31:01-bound peptides at p1, p2, p3 and pΩ are displayed after treatment with either CBZ (red) or 11-epoxide (EPX) (green) for low binding peptides (bright colors) and high binding peptides (dark colors).

CBZ treatment might alter peptide presentation through intervening in the proteome by either affecting the expression profile of proteins, therefore altering the available HLA ligands, or by manipulating components of the peptide loading complex that has a major influence on peptide presentation. Peptide motifs following treatment with both drugs were evaluated in *LCL721.221* cells. Treatment with CBZ had no effect on low binding peptides, but on high binding peptides at the auxiliary anchors p1 and p3 (Figure 3). Treatment with EPX did not have an effect on AA frequencies. In detail, the strong peptide anchor at pΩ of A*31:01 was not influenced under treatment with CBZ or EPX. With a frequency of 80% (w/CBZ) and 75% (w/EPX) the anchor AA Arginine in low binding peptides was unchanged compared to 82% (w/o drug). In high binding peptides Arginine dominated at pΩ in 93% (w/CBZ) or 71% (EPX) compared to 84% (w/o drug). The frequency of Lysine at p1 and p3 of A*31:01-high binding peptides was increased by 25% and 20%, respectively, following CBZ treatment. For low binding peptides no shift in AA frequencies was observed at these positions. P2 was not affected by treatment with CBZ.

Treatment with either drug did not affect the length of the peptides presented by A*31:01 (see Figure S3). The majority of peptides were 9 mers (w/o drug 57%, w/CBZ 56%, w/EPX 42%), followed by 11-mers (w/o drug 16%, w/CBZ 17%, w/EPX 19%).

### 3.5. CBZ Binds to sA*31:01 Heavy Chain

**Figure 4:**
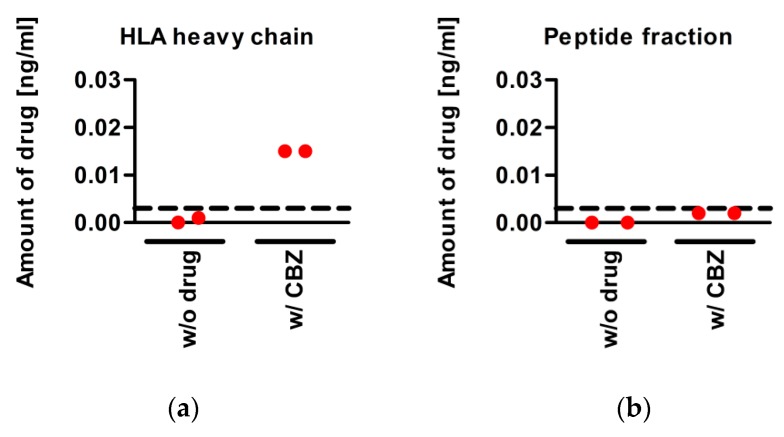
CBZ is detected after purification of the trimeric HLA molecules and further separation into (**a**) the retentate containing the HLA heavy and light chain and (**b**) the peptide fraction.

In order to understand the alteration of peptide presentation by HLA-A*31:01 induced through CBZ treatment, the ability of the drug to bind to the HLA heavy chain was measured. *LCL721.221* cells expressing sA*31:01 were treated with CBZ, the functional sHLA molecules were purified via affinity chromatography. Using mass spectrometry, the eluate comprising the trimeric molecules was analyzed to detect the presence of CBZ. Separation of low and high binding peptides and detection of CBZ revealed that the drug remained in the retentate composed of HLA heavy and light chain (Figure 4). The flow through including the peptides did not contain CBZ. This was seen after separation of low peptides as well as after TFA-mediated dissolving and separation of high binding peptides.

### 3.6. Impact of CBZ Treatment on Protein Expression of Recombinant B Cells

To understand the effect of drug treatment on the proteome of the cellular system, proteome analyses were performed.

**Figure 5:**
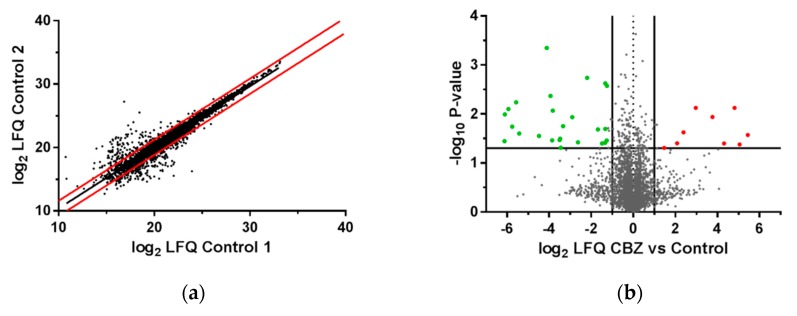

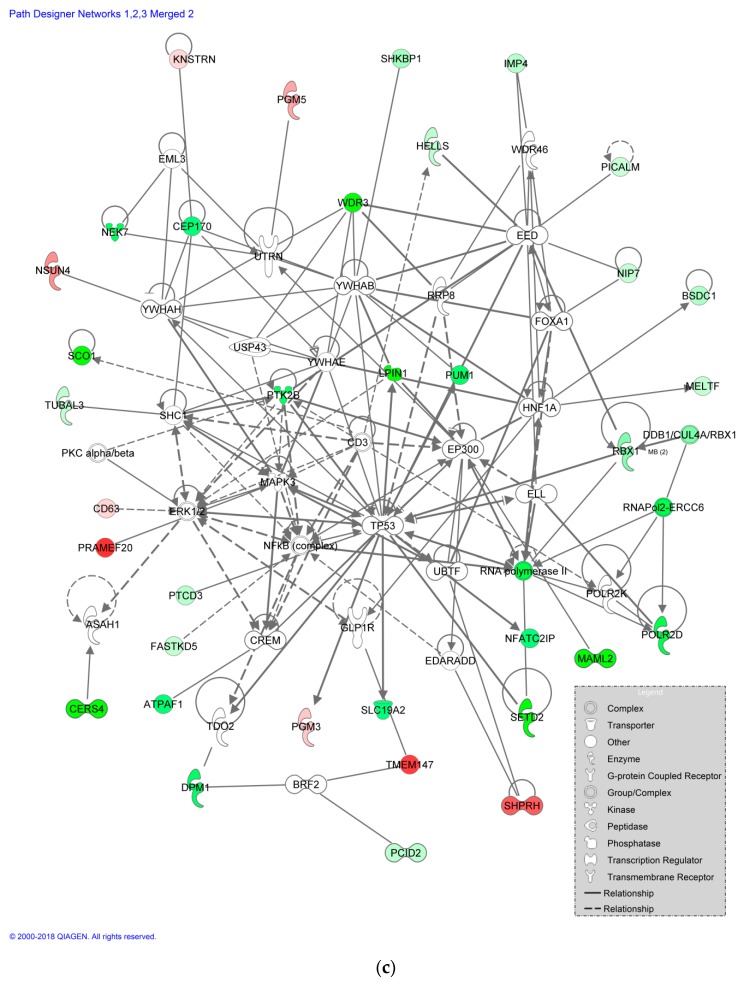
Mass spectrometric analysis of the proteome of *LCL721.221* cells expressing sA*31:01 (**a**) LFQ intensities spread of the proteome for untreated recombinant B-LCL cells transduced with sHLA-A*31:01. Protein intensity ratios of LC-MS analyzed untreated control replicates, depicted as log2-values, were plotted against each other. Regulation limits were determined through 95% confidence range (red lines) and set to log2 ± 1.2; (**b**) Protein abundance after CBZ treatment. Results are shown as a volcano plot. Protein abundance of two independent replicates is plotted as log2 value against the negative decadic logarithm of the *p*-values. Proteins were regarded as regulated from factor 1.2 and *p*-value < 0.05. Significantly upregulated proteins due to CBZ treatment are shown in red and downregulated proteins in green; (**c**) Network analysis for up- and downregulated protein groups following CBZ treatment. Upregulated proteins are illustrated in red, downregulated proteins are illustrated in green, not colored proteins were added by the IPA algorithm. High confident interactions are symbolled by a continuous line; medium confident interactions are symbolled by a dashed line.

*LCL721.221* cells expressing sA*31:01 were treated with CBZ. MS-based proteomic analysis revealed 5329 protein groups of which 4188 could be quantified. Proteins significantly (*p*-value < 0.05) regulated and at least altered by factor log2 ± 1.2 were regarded as regulated (Figure 5a). Due to CBZ treatment and considering the previously defined parameters, 9 of 4188 quantified proteins were significantly upregulated, whereas 25 were significantly downregulated (Figure 5b). As visualized in Figure 5c, interactions that to date are known to comprise the proteins down our upregulated by CBZ treatment are shown. All upregulated proteins and the 10 strongest downregulated proteins are listed in Table 2 and Table 3. After CBZ treatment the PRAME family member 20 (PRAMEF20) was the protein the strongest significantly upregulated. PRAMEF20 is involved in negative regulation of apoptotic process, cell differentiation and DNA-templated transcription, as well as in positive regulation of cell proliferation. Other significantly upregulated proteins were the E3 ubiquitin-protein ligase SHPRH, involved in DNA repair, and 5-methylcytosine rRNA methyltransferase NSUN4 that is involved in ribosome assembly and final biogenesis. The phosphatidate phosphatase LPIN1 involved in fatty acid metabolism was found to be the strongest downregulated protein after CBZ treatment of sA*31:01 expressing recombinant *LCL721.221* cells. Besides, the mastermind-like protein 2 (MAML2) acting as transcriptional coactivator of NOTCH proteins was significantly downregulated. The protein-tyrosine kinase 2-beta (PTK2B) was also identified as significantly downregulated. Due to its kinase activity PTK2B is affecting various biological processes as for example the reorganization of the actin cytoskeleton, adaptive immune response or mediation of the response to cellular stress.

### 3.7. Impact of EPX Treatment on Protein Expression of Recombinant B Cells

**Figure 6:**
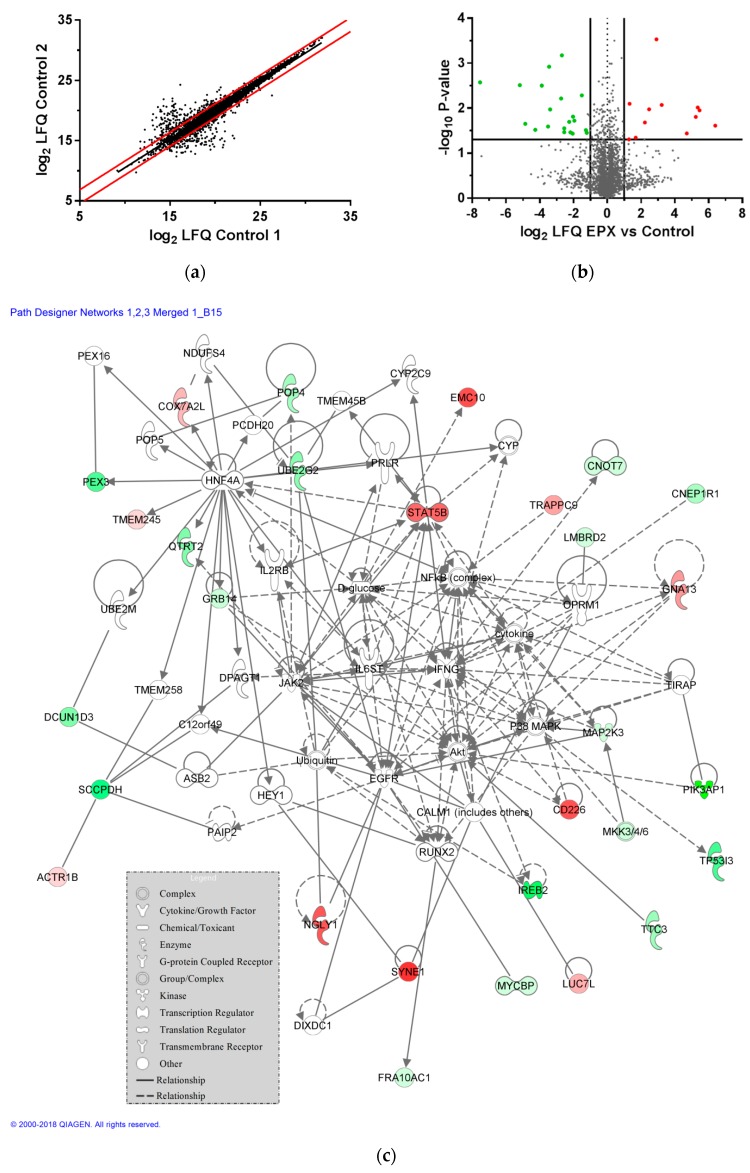
Mass spectrometric analysis of the proteome of *LCL721.221* cells expressing sB*15:02: (**a**) LFQ intensities spread of the proteome for untreated recombinant B-LCL cells transduced with sHLA-B*15:02. Protein intensity ratios of LC-MS analyzed untreated control replicates, depicted as log2-values, were plotted against each other. Regulation limits were determined through 95% confidence range (red lines) and set to log2 ± 1.1; (**b**) Protein abundance after EPX treatment. Results are shown as a volcano plot. Protein abundance of two independent replicates is plotted as log2 value against the negative decadic logarithm of the *p*-values. Proteins were regarded as regulated from factor 1.1 and *p*-value < 0.05. Significantly upregulated proteins due to EPX treatment are shown in red and downregulated proteins are shown in green; (**c**) Network analysis for up- and downregulated protein groups following EPX treatment. Upregulated proteins are illustrated in red, downregulated proteins are illustrated in green, non-colored proteins were added by the IPA algorithm. High confident interactions are symbolled by a continuous line; medium confident interactions are symbolled by a dashed line.

5360 protein groups were identified in the proteome analysis of the *LCL721.221* cells expressing sHLA-B*15:02, from which 4232 could be quantified. Taking into account the value spread of the proteome of untreated cells, solely proteins significantly regulated (*p*-value < 0.05) and at least altered by factor log2 ± 1.1 were rated as regulated (Figure 6a). Thus, 14 of 4232 quantified proteins were found significantly upregulated under EPX treatment, while 18 proteins were significantly downregulated (Figure 6b). The network in Figure 6c illustrates all currently known interactions involving the proteins identified as influenced in their expression levels by drug treatment. The 10 strongest up-/downregulated proteins are shown in Table 4 and Table 5. Nesprin-1 (SYNE1) that is involved in formation of networks between organelles and the actin cytoskeleton had the expression level the most upregulated. As well, CD226 antigen (CD226) was significantly upregulated. This cell surface molecule is involved in inflammatory regulation including intercellular adhesion and lymphocyte signaling. The transcription factor signal transducer and activator of transcription 5B (STAT5B) possessing a strong effect on signal transduction and activation of transcription in response to cytokines, was found significantly upregulated. Playing a likewise part in signal transduction, the trafficking protein particle complex subunit 9 (TRAPPC9) was highly regulated.

The most down regulated protein after treatment of the *LCL721.221* cells expressing sB*15:02 with EPX was the phosphoinositide 3-kinase adapter protein 1 (PIK3AP1). As part of the (PI3K)-Akt signaling pathway, it is of importance in B cell development. Additionally, PIK3AP1 is involved in toll-like receptor signaling and thereby prevents inflammatory cytokine production. The quinone oxidoreductase PIG3 (TP53I3) that was found to be significantly downregulated as well is thought to be involved in formation of reactive oxygen species.

## 4. Discussion

Type B ADRs are potentially life-threatening and dose-independent complications of medication appearing to be idiosyncratic [8]. Although it has been shown that ADRs in general often may be avoidable [2], unraveling the molecular mechanism of type B ADRs could allow safe medication. Associations between such ADRs and HLA alleles have been described for several drugs. For HLA-B*57:01-associated abacavir hypersensitivity the avoidance of treatment with abacavir in HLA-B*57:01-positive patients prevents an immune reaction triggered by abacavir binding to the PPR and therefore altering the repertoire of presented peptides [24,25].

In case of CBZ-mediated ADRs the molecular mechanism has not been fully understood, yet. Since CBZ-mediated ADRs are associated not only with HLA-B*15:02, but also with HLA-A*31:01, it is important to first understand the mechanistic basis of the HLA molecule functions. Both HLA molecules are linked to different clinical outcomes in CBZ-mediated ADRs. HLA-B*15:02 is associated with severe SJS and TEN, whereas carriers of HLA-A*31:01 develop mainly milder symptoms including MPE and DRESS [37,47,48,72]. Underlying those differences in their clinical picture, SJS/TEN emerge after a median of 15 days, while DRESS appears after a median of 29 days [34].

We previously demonstrated that EPX, the main metabolite of CBZ binds to the peptide binding groove and alters the peptides bound to HLA-B*15:02 [51]. This observation implies that the metabolization of CBZ and the binding of its main metabolite EPX might fundamentally impact on the dramatic clinical outcomes for B*15:02 positive patients following CBZ administration. The fast emerge of SJS/TEN is clearly explicable by the short CBZ-half lifetime of about 36 h in most patient cases. The metabolization occurs up to 97% hepatic. EPX can be detected in the blood system (serum or plasma), enters the antigen-presenting cells, binds to the HLA-B*15:02 heavy chain prior to peptide selection and this EPX/peptide/HLA-B*15:02 complexes trigger GvHD-like T-cell reactions [51].

For HLA-A*31:01, little has been evaluated until now. T cell clones generated from a HLA-A*31:01-positive patients suffering from CBZ-mediated DRESS were identified as CBZ-specific HLA-A*31:01-restricted CD8^+^ T cells as well as HLA-DRB1*04:04-restricted CD4^+^ T cells [57].

To understand functional differences in peptide recruitment between the both alleles, we analyzed their TPN-dependency. In its function of stabilizing the HLA complex during peptide loading and catalyzing the substitution of low binding with high binding peptides TPN plays a major role in peptide acquisition [73]. We could observe that sA*31:01 selects and presents peptides independently of TPN, while sB*15:02 was not able to form stable functional pHLA complexes in the absence of TPN. This functional dissimilarity between both alleles along with their different ethnic distribution and diverse clinical outcomes reveals that CBZ-hypersensitivity for HLA-A*31:01 and for HLA-B*15:02 patients are two distinct diseases.

Peptide presentation of membrane-bound HLA-A*31:01 was already examined by Abelin et al. [52] in *LCL721.*221 cells, but low and high binding peptides were not inspected separately. In general, the results were comparable with ours in *LCL721.*221 cells expressing sA*31:01. We found sA*31:01 was presenting mainly 9 mers, but the amount of 11 mers was increased compared to other HLA class I alleles. This phenomenon was seen for low, as well as for high binding peptides. Peptides presented by sA*31:01 are strongly anchored by Arginine at pΩ. In *LCL721.*22*0* cells, where peptides were acquired without the assistance of TPN Arginine could be as well observed to anchor the peptides at pΩ.

These results represent a similar binding motif of low and high binding peptides in TPN-negative *LCL721.*22*0* cells. Contrarily, in TPN-positive *LCL721.221* cells, the most frequent AA at p2 in high binding peptides was Threonine, in low binding peptides no anchor or auxiliary anchor could be detected. This leads to the suggestion, that high binding peptides have a TPN-dependent p2 anchor, whereas in the absence of TPN high binding peptides resemble low binding peptides. Peptide recruitment of TPN-independent HLA-A*31:01 is clearly a very sensitive and perfectly balanced biophysical procedure; unwanted cellular auto-immune reactions have to be avoided.

It is still unknown if CBZ or its main metabolite EPX is the main trigger of the immunological reaction for HLA-A*31:01 positive patients.

We analyzed the peptides presented by sA31:01 and compared them with sA*31:01-derived peptides following CBZ or EPX exposure. We could clearly observe an increased frequency of Lysine at p1 and p3 of high binding peptides after treatment with CBZ, whereas other positions and low binding peptides remained unaltered. The pΩ anchor remained consistent. Treatment with EPX did not affect AA frequencies. The length of the presented peptides was not affected by drug treatment. This was leading to the suggestion that binding of the drug to the HLA peptide binding groove generates a different N-terminal peptide anchor in comparison to peptides recruited without drug. This suggestion is supported by the observation that also the frequency of Arginine at pΩ was slightly increased from 84% to 93% in high binding peptides after CBZ treatment, but not in low binding peptides. Nevertheless, these results do not clearly prove the hapten model or the altered repertoire model, although an alteration in the peptide binding motive was observed.

In order to detect either binding of the drug to the HLA heavy chain or to the peptides, we performed mass spectrometric detection of CBZ on the drug-treated purified molecules. CBZ could be precisely found to be bound to the HLA heavy chain, pointing towards the altered repertoire model in this disease. Although the exact location of CBZ on the HLA heavy chain remains unclear, we could show that binding of the drugs leads to increased frequencies of the preferred AAs at auxiliary anchors p1 and p3.

Contrarily, HLA-B*15:02 has a diverging peptide binding motive including a nonpolar aromatic pΩ anchor after treatment with EPX but not CBZ [24,50,51]. Binding of the main metabolite into the F pocket of HLA-B*15:02 affects the shape of the PBR and impedes binding of non-polar aromatic anchor AA Tyrosine.

These observations underline the fact that HLA-A*31:01 and HLA-B*15:02 differ substantially in their function of presenting peptides to immune cells, as well as in their molecular mechanism triggering CBZ-mediated ADRs.

Yet, both diseases have their TPN-dependent alteration of peptides in common, since EPX is altering peptide presentation of the TPN-dependent allele HLA-B*15:02 and CBZ of HLA-A*31:01 restricted high binding peptides acquired in the presence of TPN but not of low binding peptides.

Since not all HLA-A*31:01-positive patients under CBZ-medication develop symptoms and considering the potential role of TPN, other factors apart from the HLA allele interacting with the drug are likely to contribute to the ADR. It is even possible that more than a single mechanism leads to the development of a CBZ-mediated ADR as seen in sulfamethoxazole hypersensitivity explained by the prohapten model and the p-i model [74].

The hapten/prohapten model, the p-i model and the altered repertoire model nicely explain the engagement of T cells in ADRs, but drugs can influence peptide presentation in additional manners (Figure 1). Before a peptide is presented, several filters have to be passed. Proteins are encoded in the genome, but they have to be expressed, degraded to peptides and the fitting peptides have to be transported to and loaded into the PBR. In the p-i model, the drug interferes with the recognition between HLA complex and TCR, peptide presentation itself is not altered. The altered repertoire model ensures after binding of a drug to the PBR where it triggers a structural alteration. Binding of a substance to a protein before degradation or a peptide before presentation is considered in the hapten/prohapten model.

However, there are additional ways of modifying the peptidome than explained by the three models. Treatment with a drug might also influence expression of later presented peptides or of molecules being involved in the process of peptide loading. Therefore, alterations of the proteome through alterations of the gene expression profile can affect available peptides for presentation by HLA-molecules. Alternatively, peptide loading itself might be modified, inducing a shift in the peptidome for example in favor of low binding peptides if TPN is hindered or down regulated. If drug treatment leads to an altered ubiquitination and cleavage, changes in the cleavage profile of the proteins will affect the quantity of potential HLA ligands. This requires a wider way of thinking for understanding the emergence of ADRs.

In order to evaluate the influence of drug treatment on the cell, we examined the effect of drug treatment on the cellular proteome. EPX treatment altered peptide features of sB*15:02 and CBZ treatment of sA*31:01, consequently the presence of a different proteomic profile seemed obvious. We analyzed the proteome of the respective cells following drug treatment.

The results illustrate specific differences in the proteome due to drug treatment. In order to verify significantly regulated protein groups (*p*-value < 0.05) as up- or downregulated, 95% confidence range regulation limits were specified and set to log2 ± 1.2 for CBZ-treated sA*31:01-expressing cells and log2 ± 1.1 for EPX-treated sB*15:02-expressing cells, as calculated based on alterations compared to untreated cells. With 4188 quantified protein groups for CBZ-treated and 4232 for EPX-treated cells the proteome coverage is equal to comparable proteomic analyses in eukaryotic systems [67]. Low dynamic regulation range and few significantly regulated proteins indicated a raise of small overall regulatory impact of the treatment. CBZ and EPX take effect by inhibiting sodium channels in mammalian central neurons not requiring large cellular alterations for their effectiveness [75]. Therefore, these results show high target specificity.

In addition, alterations of proteins might also be on a posttranslational level, including phosphorylation and ubiquitination, and thus were not detectable here.

Previous results and this study are pointing towards the altered repertoire model for both alleles associated with CBZ-mediated ADRs. For HLA-I molecules the peptide-loading complex includes proteins such as TAP1, TAP2, TPN, calreticulin and ERp57 [76]. All of those proteins were quantifiable, but not significantly regulated. This shows, neither CBZ nor EPX had an immediate impact on the abundance of peptide-loading complex proteins. Nevertheless, the influence might be not MS-detectable.

CBZ treated cells showed a strong upregulation of SHPRH, a protein directly involved in ubiquitination, causing a high ubiquitinylation rate, possibly due to cellular stress or an increase of proteasome-based degradation [77]. However, no decrease in viability of the recombinant cell lines could be observed after treatment with CBZ, neither on cellular level (data not shown) nor in the proteomic analyses. This might be due to the strongly regulated antiapoptotic protein PRAMEF20 and MAML2 [78,79]. Furthermore, translational activity and metabolic activity are not reduced, as proteins such as NSUN4 are upregulated and LPIN1 are downregulated [80,81]. Along with the downregulation of PTK2B, these data suggest a complex reaction to CBZ, involving different mechanisms stabilizing the cell viability, while increasing the ubiquitinylation activity [82]. This is further supported by network analysis showing proteins from diverse signaling pathways to be regulated.

After EPX treatment proteins involved in cytoskeleton arrangement or protein trafficking (TRAPPC9, SYNE1) were found to be significantly upregulated [83,84]. Together with upregulated proteins linked to inflammatory regulation, such as CD226 antigen, and STAT5B, as major regulator of signal transduction and transcriptional activation in response to cytokines, this might imply a potential regulation of receptor composition and immune related cell surface proteins or secreted factors [85,86]. Due to downregulation of PIK3AP1, its negative regulatory influence on inflammatory cytokine production was decreased, supporting hints of EPX being an activator of cytokine release [87]. Further analyses of the secretome and cell surface receptors are required to verify these results. Especially the secreted cytokines might be of interest due to possible paracrine effects on the HLA-I presented peptide composition as previously described [24].

The presented data of the proteomic analyses of CBZ- and EPX-treated human lymphoblastoid B cell lines present an initial screening and further measurements and exploration of existing data might provide a more detailed awareness. These results give a first insight into possible regulations but require further functional verification. To specify the impact of drug treatment, further investigations could be conducted, varying time points and concentrations of medication. Moreover, the measured protein abundances could be supplemented with an analysis of the phospho-proteome to examine the activity status of signal pathways and transcriptions factors above protein level.

Taken together, the presented data allow a first glance into the fine, but complex regulatory impact of CBZ or EPX. This study allows for the first time an overview of possible connections of molecular mechanism and macroscopic observations in a non-hypothesis driven, screening approach. The presented candidate proteins show strong regulations in proteomic data and might be interesting candidates for further investigations.

Although the proteome analysis led to new findings, it did not unravel the molecular mechanism of CBZ hypersensitivity in HLA-A*31:01-positive patients. Results from the peptide analysis and the examination of the proteome underline the fact that HLA-A*31:01- and B*15:02-associated CBZ-hypersensitivity are two distinct diseases. HLA-A*31:01-positive patients experiencing a CBZ-mediated ADR, develop an immune response triggered by presentation of an altered self-peptide repertoire with high affinity while unaltered self-peptides of low affinity might be co-presented; whereas EPX triggers a reaction in B*15:02-positive individuals presenting altered peptides in a TPN-dependent manner.

The molecular mechanism in HLA-A*31:01-positive patients is not fully understood; however, it might be explained by the described alterations of the proteome including increased ubiquitination. In combination with binding of the drug to the HLA heavy chain and the concurrent presentation of unmodified low binding peptides and altered presentation of high binding peptides, the accessible surface for a TCR and potentially the half-life time of the peptide-HLA complexes might change. These processes could moderate the immune response leading to less severe conditions of MPE or DRESS. In HLA-B*15:02-positive patients, mainly altered high binding peptides are presented due to binding of EPX in the F pocket [51]. Those peptides have a high affinity to the HLA molecules, form stable pHLA complexes and provoke a strong T cell response. That might explain the life-threatening complications of SJS/TEN. Therefore, when evaluating the risk of CBZ-mediated ADRs, it is important to differentiate between the two alleles.

As HLA class I molecules are the primary interaction partner for CD8^+^ T cells, further considerations that take interactions with the individual T cell receptors into account may help characterize new risk factors and allow for a thorough comprehension of their onset.

By understanding HLA-B*15:02- and HLA-A*31:01-associated CBZ-mediated ADRs on a cellular, molecular and mechanistic level might introduce new ways of optimizing medication of patients preventing potentially fatal and expensive complications.

## 5. Conclusions

This study demonstrates for the first time the extremely complex concurrence between carbamazepine hypersensitivity, genomics, proteomics and peptidomics to support personalized and safety drug treatment.

## Figures and Tables

**Table 1:** Elution solutions for fractionation of digest sample.

Fraction No.	Acetonitrile (%)	Triethylamine (%)
1	7.5	0.1
2	12.5	0.1
3	15	0.1
4	17.5	0.1
5	50	0.1

**Table 2:** Strongest upregulated proteins after CBZ treatment.

Protein Name	Gene Code	log_2_ Regulation	*p*-Value
PRAME family member 20/21	PRAMEF20	5.44	0.027
Transmembrane protein 147	TMEM147	5.05	0.042
Small EDRK-rich factor 2	SERF2	4.81	0.008
E3 ubiquitin-protein ligase SHPRH	SHPRH	4.31	0.040
Zinc finger protein Helios	IKZF2;ZNFN1A2	3.76	0.012
5-methylcytosine rRNA methyltransferase	NSUN4	2.97	0.007
Phosphoglucomutase-like protein 5	PGM5	2.39	0.024
Protein kinase C-binding protein 1	ZMYND8	2.08	0.040
Phosphoacetylglucosamine mutase	PGM3	1.47	0.049

**Table 3:** Strongest downregulated proteins after CBZ treatment.

Protein Name	Gene Code	log_2_ Regulation	*p*-Value
Phosphatidate phosphatase LPIN1	LPIN1	−6.12	0.036
Ceramide synthase 4	CERS4	−6.11	0.010
Histone-lysine N-methyltransferase SETD2	SETD2	−5.94	0.008
Mastermind-like protein 2	MAML2	−5.76	0.018
Protein SCO1 homolog, mitochondrial	SCO1	−5.58	0.006
WD repeat-containing protein 3	WDR3	−5.42	0.025
DNA-directed RNA polymerase II subunit RPB4	POLR2D	−4.49	0.028
Serine/threonine-protein kinase Nek7	NEK7	−4.12	<0.001
Protein-tyrosine kinase 2-beta	PTK2B	−3.94	0.004
Dolichol-phosphate mannosyltransferase subunit 1	DPM1	−3.86	0.034

**Table 4:** Strongest upregulated proteins after EPX treatment.

Protein Name	Gene Code	log_2_ Regulation	*p*-Value
Nesprin-1	SYNE1	6.39	0.025
ER membrane protein complex subunit 10	EMC10	5.46	0.011
CD226 antigen	CD226	5.35	0.010
Peptide-N(4)-(N-acetyl-beta-glucosaminyl)asparagine amidase	NGLY1	5.24	0.016
Signal transducer and activator of transcription 5B	STAT5B	4.70	0.036
Guanine nucleotide-binding protein subunit alpha-13	GNA13	3.22	0.008
Trafficking protein particle complex subunit 9	TRAPPC9	2.91	<0.001
Putative RNA-binding protein Luc7-like 1	LUC7L	2.47	0.011
Cytochrome c oxidase subunit 7A-related protein, mitochondrial	COX7A2L	2.23	0.021
ESF1 homolog	ESF1	1.68	0.045

**Table 5:** Strongest downregulated proteins after EPX treatment.

Protein Name	Gene Code	log_2_ Regulation	*p*-Value
Phosphoinositide 3-kinase adapter protein 1	PIK3AP1	−7.53	0.003
Spermidine/spermine N(1)-acetyltransferase-like protein1	SATL1	−5.18	0.003
Iron-responsive element-binding protein 2	IREB2	−4.85	0.022
Protein FAM177A1	FAM177A1	−4.26	0.030
Saccharopine dehydrogenase-like oxidoreductase	SCCPDH	−3.88	0.003
Methylsterol monooxygenase 1	MSMO1	−3.51	0.026
Quinone oxidoreductase PIG3	TP53I3	−3.45	0.001
Peroxisomal biogenesis factor 3	PEX3	−3.38	0.011
DCN1-like protein 3	DCUN1D3	−2.73	0.006
Queuine tRNA-ribosyltransferase subunit	QTRTD1	−2.69	0.001

## Supplementary Materials

The following are available online at https://www.mdpi.com/1999-4923/11/10/536/s1, Figure S1. Solvent gradient for LC-MS analysis of peptides. Figure S2. Production of sA*31:01 is independent of TPN, whereas sB*15:02 requires TPN. The TPN+ cell line *LCL721.221* and the TPN- cell line *LCL721.220* were lentivirally transduced with vectors encoding for sA*31:01 or B*15:02. The concentration of sHLA in cell culture supernatant of untransduced and transduced *LCL721.221* and *LCL721.220* cells was determined via HLA class I specific ELISA. Figure S3. Length of presented peptides after drug treatment. The length of peptides bound to sA*31:01 without (blue) treatment and after treatment with CBZ (red) and EPX (green). Table S1. Amino acid frequencies in sA*31:01-restricted low binding peptides produced in *LCL721.221* cells. Table S2. Amino acid frequencies in sA*31:01-restricted high binding peptides produced in *LCL721.221* cells. Table S3. Amino acid frequencies in sA*31:01-restricted low binding peptides produced in *LCL721.220* cells. Table S4. Amino acid frequencies in sA*31:01-restricted high binding peptides produced in *LCL721.220* cells. Table S5. Amino acid frequencies in sA*31:01-restricted low binding peptides produced in LCL721.221 cells treated with EPX. Table S6. Amino acid frequencies in sA*31:01-restricted high binding peptides produced in *LCL721.221* cells treated with EPX. Table S7. Amino acid frequencies in sA*31:01-restricted low binding peptides produced in *LCL721.221* cells treated with CBZ. Table S8. Amino acid frequencies in sA*31:01-restricted high binding peptides produced in *LCL721.221* cells treated with CBZ.
