# Supplementary material for: The Mechanistic Differences in HLA-Associated Carbamazepine Hypersensitivity
Source: Pharmaceutics. 2019 Oct 15;11(10):536. doi: 10.3390/pharmaceutics11100536 (PMC6835980; doi:10.3390/pharmaceutics11100536)
Supplement: Supplementary file 1 [file pharmaceutics-11-00536-s001.pdf]

pharmaceutics

Supplementary Materials: The Mechanistic Differences in HLA-Associated Carbamazepine Hypersensitivity

Gwendolin S. Simper, Lareen S. Gräser, Alexander A. Celik, Joachim Kuhn, Heike Kunze-Schumacher, Gia-Gia T. Hò, Rainer Blasczyk, Andreas Pich and Christina Bade-Doeding

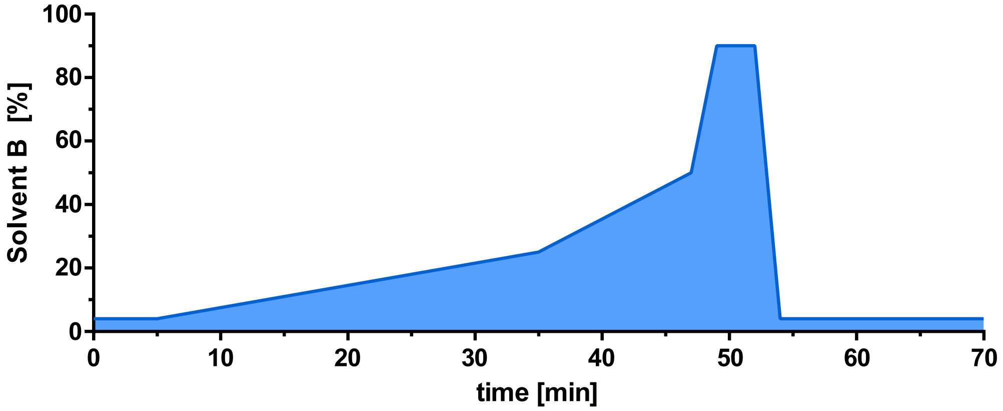

Figure S1. Solvent gradient for LC-MS analysis of peptides.

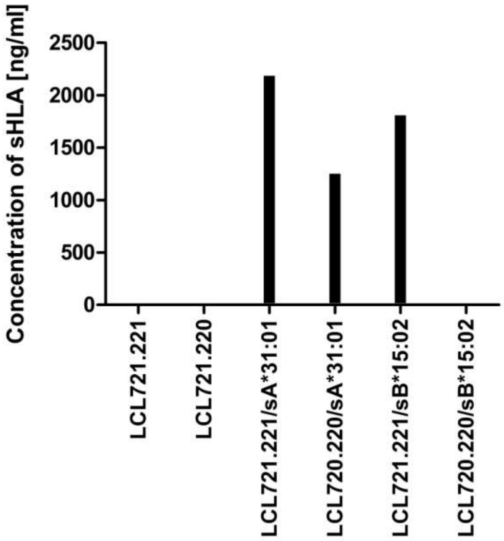

Figure S2. Production of sA*31:01 is independent of TPN, whereas sB*15:02 requires TPN. The TPN+ cell line *LCL721.221* and the TPN- cell line *LCL721.220* were lentivirally transduced with vectors encoding for sA*31:01 or B*15:02. The concentration of sHLA in cell culture supernatant of untransduced and transduced *LCL721.221* and *LCL721.220* cells was determined via HLA class I specific ELISA.

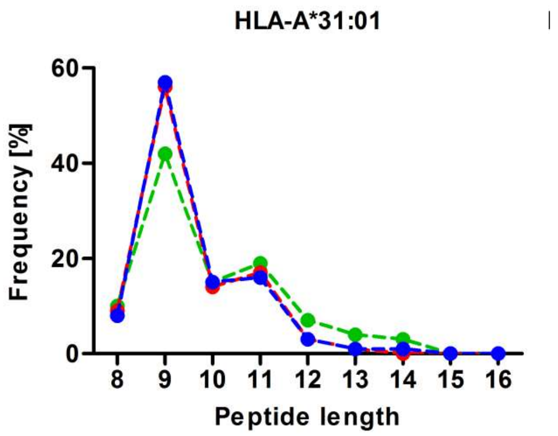

Figure S3. Length of presented peptides after drug treatment. The length of peptides bound to sA*31:01 without (blue) treatment and after treatment with CBZ (red) and EPX (green).

Table S1. Amino acid frequencies in sA*31:01-restricted low binding peptides produced in *LCL721.221* cells.

AA	P1	P2	P3	P4	P5	P6	P7	P8	P9	P10	P11	PΩ
H	3%	0%	2%	1%	2%	4%	6%	4%	2%	2%	1%	1%
K	25%	2%	24%	7%	9%	7%	6%	9%	6%	10%	11%	9%
R	20%	1%	4%	1%	1%	1%	1%	7%	55%	31%	67%	82%
D	1%	1%	2%	7%	3%	2%	2%	2%	1%	1%	0%	0%
E	1%	0%	2%	11%	4%	5%	5%	7%	2%	2%	1%	0%
C	0%	0%	0%	0%	0%	0%	0%	0%	0%	0%	0%	0%
N	1%	2%	10%	5%	5%	3%	4%	6%	3%	3%	1%	0%
P	0%	1%	1%	8%	9%	3%	4%	4%	3%	4%	2%	1%
Q	4%	10%	12%	7%	6%	5%	7%	9%	3%	8%	0%	1%
S	11%	15%	7%	9%	9%	6%	7%	7%	5%	6%	3%	0%
T	3%	17%	3%	8%	5%	7%	9%	5%	3%	3%	2%	0%
A	14%	9%	7%	10%	8%	7%	10%	9%	3%	5%	2%	1%
G	5%	7%	4%	10%	8%	9%	5%	6%	3%	6%	2%	1%
I	3%	4%	4%	3%	7%	8%	6%	4%	2%	2%	0%	0%
L	2%	8%	8%	6%	8%	13%	8%	8%	3%	6%	3%	2%
M	1%	2%	2%	1%	0%	1%	1%	1%	1%	2%	0%	0%
V	5%	17%	3%	5%	10%	11%	15%	9%	4%	6%	1%	1%
F	1%	1%	3%	1%	3%	4%	2%	1%	1%	1%	0%	0%
W	0%	0%	0%	0%	0%	1%	0%	0%	0%	0%	0%	0%
Y	0%	3%	2%	1%	2%	2%	3%	2%	1%	1%	0%	0%

Table S2. Amino acid frequencies in sA*31:01-restricted high binding peptides produced in *LCL721.221* cells.

AA	P1	P2	P3	P4	P5	P6	P7	P8	P9	P10	P11	PΩ
H	2%	0%	0%	0%	1%	3%	2%	2%	1%	1%	0%	0%
K	19%	2%	21%	5%	7%	8%	4%	6%	4%	6%	10%	8%
R	9%	1%	2%	0%	1%	1%	2%	7%	59%	32%	59%	84%
D	0%	1%	2%	6%	3%	2%	1%	2%	0%	2%	1%	0%
E	0%	1%	2%	10%	4%	3%	4%	8%	1%	2%	4%	0%
C	1%	0%	0%	0%	0%	0%	0%	0%	0%	0%	0%	0%
N	2%	2%	13%	6%	6%	3%	4%	9%	3%	4%	1%	0%
P	0%	1%	2%	9%	9%	4%	5%	5%	3%	4%	0%	1%
Q	5%	10%	15%	7%	4%	4%	6%	10%	3%	6%	1%	1%
S	11%	19%	9%	12%	11%	7%	10%	9%	5%	6%	1%	1%
T	7%	20%	3%	10%	9%	6%	10%	5%	3%	3%	4%	0%
A	21%	6%	8%	13%	8%	8%	11%	10%	4%	8%	4%	2%
G	9%	9%	5%	12%	7%	9%	4%	6%	2%	6%	4%	0%
I	5%	4%	2%	1%	6%	7%	7%	2%	2%	3%	0%	0%
L	2%	5%	6%	2%	9%	12%	5%	7%	4%	7%	7%	1%
M	1%	1%	1%	0%	1%	1%	1%	1%	0%	4%	0%	0%
V	6%	17%	4%	4%	12%	13%	17%	7%	4%	6%	0%	1%
F	0%	0%	2%	0%	2%	4%	2%	1%	1%	0%	0%	0%
W	0%	0%	0%	1%	0%	0%	0%	1%	0%	0%	0%	0%
Y	0%	1%	2%	2%	1%	3%	4%	2%	1%	1%	0%	0%

Table S3. Amino acid frequencies in sA*31:01-restricted low binding peptides produced in *LCL721.220* cells.

AA	P1	P2	P3	P4	P5	P6	P7	P8	P9	P10	P11	PΩ
H	4%	1%	0%	2%	1%	2%	1%	1%	1%	1%	0%	0%
K	12%	0%	21%	8%	12%	6%	7%	3%	7%	5%	13%	10%
R	31%	0%	5%	1%	1%	4%	3%	5%	35%	22%	42%	86%
D	0%	1%	1%	16%	3%	7%	2%	1%	0%	1%	0%	0%
E	1%	0%	1%	12%	5%	5%	3%	8%	4%	1%	1%	1%
C	0%	0%	0%	0%	0%	0%	0%	0%	0%	0%	0%	0%
N	1%	1%	5%	5%	3%	4%	8%	3%	4%	5%	1%	0%
P	2%	1%	2%	12%	10%	3%	5%	5%	4%	1%	0%	0%
Q	1%	4%	5%	3%	0%	3%	1%	6%	1%	5%	7%	0%
S	12%	6%	3%	6%	5%	5%	6%	8%	4%	9%	4%	0%
T	5%	16%	1%	5%	1%	5%	10%	5%	8%	4%	3%	0%
A	10%	5%	2%	5%	8%	6%	6%	7%	4%	10%	3%	0%
G	5%	6%	3%	8%	10%	10%	5%	12%	7%	8%	7%	1%
I	6%	5%	8%	3%	9%	6%	12%	6%	6%	3%	4%	1%
L	2%	9%	16%	3%	7%	10%	10%	11%	4%	10%	7%	1%
M	1%	5%	3%	0%	1%	2%	1%	1%	1%	2%	3%	0%
V	8%	28%	4%	5%	12%	8%	13%	9%	6%	10%	4%	0%
F	1%	5%	8%	2%	6%	8%	5%	3%	3%	2%	0%	1%
W	0%	0%	2%	1%	2%	2%	1%	1%	0%	0%	0%	0%
Y	0%	8%	8%	2%	5%	4%	2%	5%	1%	0%	1%	0%

Table S4. Amino acid frequencies in sA*31:01-restricted high binding peptides produced in *LCL721.220* cells.

AA	P1	P2	P3	P4	P5	P6	P7	P8	P9	P10	P11	PΩ
H	5%	0%	2%	0%	0%	0%	2%	2%	3%	4%	0%	0%
K	7%	0%	28%	12%	16%	21%	7%	5%	13%	8%	11%	5%
R	33%	0%	2%	0%	2%	5%	5%	12%	38%	17%	79%	95%
D	0%	2%	0%	14%	0%	5%	5%	0%	0%	0%	0%	0%
E	0%	0%	0%	9%	0%	2%	2%	12%	3%	0%	0%	0%
C	0%	0%	0%	0%	0%	0%	0%	0%	0%	0%	0%	0%
N	0%	5%	2%	5%	0%	0%	9%	2%	0%	0%	0%	0%
P	2%	0%	2%	12%	9%	2%	7%	5%	0%	0%	0%	0%
Q	5%	5%	5%	0%	0%	0%	2%	5%	5%	8%	0%	0%
S	12%	5%	2%	5%	7%	2%	7%	5%	0%	21%	5%	0%
T	9%	16%	0%	5%	5%	7%	2%	7%	8%	4%	0%	0%
A	2%	0%	5%	5%	12%	5%	9%	5%	3%	4%	0%	0%
G	5%	9%	7%	12%	9%	12%	2%	5%	5%	13%	5%	0%
I	2%	12%	14%	5%	12%	5%	16%	7%	3%	0%	0%	0%
L	5%	12%	12%	5%	9%	16%	2%	16%	8%	8%	0%	0%
M	2%	2%	2%	0%	0%	2%	0%	2%	3%	0%	0%	0%
V	9%	26%	5%	7%	9%	7%	14%	9%	8%	13%	0%	0%
F	2%	0%	7%	0%	5%	7%	5%	0%	3%	0%	0%	0%
W	0%	2%	0%	0%	5%	0%	0%	0%	0%	0%	0%	0%
Y	0%	5%	5%	7%	0%	2%	2%	2%	0%	0%	0%	0%

Table S5. Amino acid frequencies in sA*31:01-restricted low binding peptides produced in LCL721.221 cells treated with EPX.

AA	P1	P2	P3	P4	P5	P6	P7	P8	P9	P10	PΩ
H	2%	0%	1%	1%	1%	3%	6%	4%	1%	0%	0%
K	24%	2%	16%	5%	8%	7%	7%	9%	7%	21%	12%
R	17%	1%	2%	2%	1%	1%	1%	8%	67%	61%	75%
D	1%	2%	2%	8%	4%	2%	2%	1%	0%	0%	0%
E	1%	1%	3%	11%	6%	5%	5%	6%	1%	1%	0%
C	0%	0%	0%	0%	0%	0%	0%	0%	0%	0%	0%
N	3%	3%	11%	4%	4%	3%	3%	7%	1%	1%	0%
P	0%	2%	2%	8%	8%	4%	3%	6%	2%	2%	1%
Q	4%	7%	10%	7%	5%	5%	9%	11%	1%	3%	2%
S	9%	14%	6%	8%	8%	3%	6%	6%	1%	1%	1%
T	3%	16%	3%	7%	5%	5%	7%	5%	2%	1%	1%
A	12%	8%	7%	8%	6%	6%	9%	6%	2%	1%	1%
G	6%	9%	4%	9%	6%	5%	2%	7%	2%	3%	2%
I	4%	4%	6%	4%	9%	10%	9%	4%	1%	0%	1%
L	4%	8%	10%	9%	11%	16%	11%	7%	5%	4%	2%
M	2%	2%	3%	1%	1%	2%	1%	1%	0%	1%	0%
V	6%	15%	4%	5%	10%	13%	14%	7%	3%	1%	1%
F	1%	2%	5%	1%	4%	5%	3%	2%	1%	1%	1%
W	0%	0%	0%	0%	1%	1%	0%	0%	0%	1%	0%
Y	1%	3%	4%	2%	3%	3%	3%	3%	1%	0%	0%

Table S6. Amino acid frequencies in sA*31:01-restricted high binding peptides produced in *LCL721.221* cells treated with EPX.

AA	P1	P2	P3	P4	P5	P6	P7	P8	P9	P10	P11	PΩ
H	3%	0%	1%	0%	1%	3%	4%	2%	2%	2%	1%	1%
K	21%	2%	21%	6%	6%	5%	6%	7%	7%	8%	14%	19%
R	17%	1%	4%	1%	1%	1%	1%	5%	41%	21%	38%	71%
D	1%	2%	2%	10%	5%	4%	3%	4%	2%	2%	3%	0%
E	1%	2%	4%	11%	6%	6%	6%	8%	2%	6%	6%	1%
C	0%	0%	0%	0%	0%	0%	0%	0%	0%	0%	0%	0%
N	2%	2%	10%	6%	6%	4%	4%	7%	3%	5%	3%	0%
P	0%	1%	2%	9%	8%	5%	4%	5%	2%	4%	1%	1%
Q	2%	7%	11%	6%	5%	5%	6%	8%	4%	8%	2%	2%
S	9%	15%	6%	9%	9%	7%	8%	7%	3%	7%	3%	1%
T	5%	18%	3%	8%	6%	6%	8%	6%	5%	3%	6%	0%
A	13%	7%	6%	10%	7%	7%	8%	9%	4%	8%	4%	1%
G	7%	9%	5%	9%	9%	9%	6%	7%	4%	6%	3%	1%
I	4%	3%	4%	3%	7%	9%	7%	4%	3%	2%	3%	0%
L	3%	7%	8%	5%	6%	12%	7%	8%	5%	5%	6%	2%
M	1%	2%	3%	0%	1%	0%	1%	1%	1%	1%	0%	0%
V	7%	17%	4%	4%	11%	9%	15%	9%	7%	8%	3%	0%
F	1%	1%	4%	2%	3%	3%	2%	2%	2%	1%	1%	0%
W	0%	0%	0%	1%	1%	1%	0%	0%	1%	0%	0%	0%
Y	1%	4%	3%	1%	2%	4%	4%	3%	2%	2%	2%	0%

Table S7. Amino acid frequencies in sA*31:01-restricted low binding peptides produced in *LCL721.221* cells treated with CBZ.

AA	P1	P2	P3	P4	P5	P6	P7	P8	P9	P10	P11	PΩ
H	2%	0%	2%	1%	2%	4%	6%	5%	2%	1%	1%	1%
K	27%	2%	27%	7%	8%	8%	7%	8%	7%	9%	14%	11%
R	19%	1%	4%	1%	1%	1%	1%	7%	54%	34%	60%	80%
D	0%	1%	2%	7%	3%	2%	3%	2%	1%	1%	1%	1%
E	1%	1%	3%	12%	5%	5%	5%	6%	2%	4%	2%	1%
C	0%	0%	0%	0%	0%	0%	0%	0%	0%	0%	0%	0%
N	2%	2%	10%	5%	4%	2%	4%	7%	3%	4%	1%	0%
P	1%	1%	1%	8%	9%	4%	4%	5%	2%	2%	0%	1%
Q	3%	9%	11%	8%	5%	5%	7%	8%	3%	6%	2%	1%
S	10%	15%	6%	10%	9%	6%	6%	8%	4%	7%	4%	0%
T	4%	20%	3%	7%	4%	8%	8%	6%	3%	4%	5%	1%
A	13%	7%	6%	10%	8%	7%	9%	8%	3%	6%	3%	1%
G	6%	7%	4%	10%	8%	9%	5%	6%	3%	8%	2%	1%
I	3%	3%	4%	3%	8%	9%	7%	4%	1%	1%	2%	0%
L	3%	7%	7%	7%	8%	12%	9%	8%	5%	6%	3%	2%
M	1%	3%	2%	0%	0%	1%	1%	1%	1%	1%	1%	0%
V	5%	19%	4%	5%	12%	13%	15%	8%	5%	5%	2%	1%
F	1%	1%	3%	0%	2%	2%	2%	1%	1%	1%	0%	0%
W	0%	0%	0%	0%	0%	0%	0%	0%	0%	0%	0%	0%
Y	0%	2%	2%	1%	1%	3%	2%	2%	1%	0%	1%	0%

Table S8. Amino acid frequencies in sA*31:01-restricted high binding peptides produced in *LCL721.221* cells treated with CBZ.

AA	P1	P2	P3	P4	P5	P6	P7	P8	P9	P10	P11	PΩ
H	0%	0%	0%	0%	4%	9%	7%	7%	4%	3%	0%	2%
K	44%	0%	42%	7%	11%	10%	14%	14%	2%	8%	11%	5%
R	21%	1%	0%	0%	0%	0%	0%	0%	51%	29%	70%	93%
D	0%	0%	0%	7%	5%	4%	1%	1%	0%	0%	0%	0%
E	0%	0%	1%	14%	1%	2%	0%	7%	2%	3%	4%	0%
C	0%	0%	0%	0%	0%	0%	0%	0%	1%	0%	0%	0%
N	0%	1%	15%	10%	4%	2%	4%	6%	4%	0%	0%	0%
P	0%	0%	0%	5%	4%	0%	1%	2%	5%	3%	0%	0%
Q	2%	6%	9%	2%	1%	2%	7%	9%	4%	5%	0%	0%
S	2%	21%	7%	15%	7%	6%	4%	7%	4%	3%	0%	0%
T	1%	22%	1%	6%	7%	2%	6%	4%	2%	3%	0%	0%
A	12%	7%	5%	6%	9%	11%	10%	10%	5%	8%	4%	0%
G	2%	5%	1%	16%	12%	5%	7%	5%	1%	13%	0%	0%
I	5%	2%	5%	1%	12%	7%	7%	4%	4%	5%	0%	0%
L	0%	9%	5%	4%	9%	19%	11%	9%	7%	3%	7%	0%
M	1%	1%	2%	0%	0%	0%	0%	4%	0%	3%	0%	0%
V	6%	22%	4%	5%	12%	15%	16%	7%	2%	13%	0%	0%
F	1%	0%	2%	1%	1%	2%	0%	0%	1%	0%	0%	0%
W	0%	0%	0%	0%	0%	0%	0%	0%	0%	0%	0%	0%
Y	0%	1%	0%	0%	0%	2%	4%	4%	0%	0%	4%	0%
